# Incremental Value of Biventricular Strain in Patients with Severe Aortic Stenosis

**DOI:** 10.3390/jcdd11030090

**Published:** 2024-03-13

**Authors:** Camille Sarrazyn, Xavier Galloo, Maria Chiara Meucci, Steele C. Butcher, Kensuke Hirsawa, Rinchyenkhand Myagmardorj, Frank van der Kley, Tine De Backer, Jeroen J. Bax, Nina Ajmone Marsan

**Affiliations:** 1 Department of Cardiology, Leiden University Medical Center, 2300ZA Leiden, The Netherlands; c.sarrazyn@lumc.nl (C.S.); x.galloo@lumc.nl (X.G.); j.j.bax@lumc.nl (J.J.B.); 2 Department of Cardiology, Ghent University Hospital, 9000 Gent, Belgium; tine.debacker@ugent.be; 3 Department of Cardiology, Free University Brussels (VUB), University Hospital Brussels (UZ Brussel), 1090 Brussels, Belgium; 4 Department of Cardiovascular Medicine, Fondazione Policlinico Universitario A. Gemelli IRCCS, 00136 Rome, Italy; 5 Department of Cardiology, Royal Perth Hospital, Perth 6000, WA, Australia; 6 Department of Cardiovascular Medicine, Tokyo Medical and Dental University, Tokyo 113-8510, Japan; 7 Faculty of Medicine and Health Sciences, Ghent University, 9000 Ghent, Belgium

**Keywords:** valvular heart disease, imaging, echocardiography, speckle-tracking echocardiography, left ventricular global longitudinal strain, right ventricular free wall strain, aortic valve stenosis, transcatheter aortic valve implantation

## Abstract

(1) Background: Left ventricular global longitudinal (LVGLS) and right ventricular free wall strain (RVFWS) demonstrated separate prognostic values in patients with severe aortic stenosis (AS). However, studies evaluating the combined assessment of LVGLS and RVFWS have shown contradictory results. This study explored the prognostic value of combining LVGLS and RVFWS in a large group of severe AS patients referred for transcatheter aortic valve implantation. (2) Methods: Patients were classified into three groups: preserved (LVGLS ≥ 15% AND RVFWS > 20%), single-ventricle impaired (LVGLS < 15% OR RVFWS ≤ 20%), or biventricular-impaired strain group (LVGLS < 15% AND RVFWS ≤ 20%). The cut-off values were based on previously published data and spline analyses. The endpoint was all-cause mortality. (3) Results: Of the 712 patients included (age 80 ± 7 years, 53% men), 248 (35%) died. The single-ventricle impaired and biventricular-impaired (vs. preserved) strain groups showed significantly lower 5-year survival rates (68% and 55% vs. 77%, respectively, *p* < 0.001). Through multivariable analysis, single-ventricle impaired (HR 1.762; 95% CI: 1.114–2.788; *p* = 0.015) and biventricular-impaired strain groups (HR 1.920; 95% CI: 1.134–3.250; *p* = 0.015) were independently associated with all-cause mortality. These findings were confirmed with a sensitivity analysis in patients with preserved LV ejection fraction. (4) Conclusions: In patients with severe AS, biventricular strain allows better risk stratification, even if LV ejection fraction is preserved.

## 1. Introduction

Aortic valve stenosis (AS) is a common valvular heart disease characterized by a chronic left ventricular (LV) pressure overload, which leads to LV hypertrophy and remodeling, and ultimately results in myocardial fibrosis and dysfunction [1,2,3]. The occurrence of LV dysfunction in patients with AS has been associated with a significantly worse prognosis, and the current guidelines therefore recommend aortic valve replacement in the case of reduced LV ejection fraction (LVEF < 50%), even in asymptomatic patients [4]. However, several studies have demonstrated that in AS patients, LV global longitudinal strain (LV GLS) is also a more sensitive indicator than LVEF to detect subclinical LV dysfunction, and when impaired, is associated with reduced survival [5,6,7,8,9]. Additionally, when the hemodynamic effects of chronic pressure overload extend to the right ventricle (RV), RV remodeling and dysfunction may occur, which have also been shown to be associated with worse outcomes after aortic valve intervention [10,11]. The role of conventional echocardiographic parameters of RV function for risk stratification in AS has been debated, while RV free wall strain (RV FWS) has consistently shown to be more sensitive in the earlier detection of RV dysfunction and to be associated with higher mortality rates [12,13,14,15,16].

A combined assessment of LV and RV strain In patients with severe AS has been performed in only few studies of which have shown contradictory results, with LV GLS and RV FWS not always independently associated with outcomes [17,18,19].

The aim of this study was therefore to assess the prognostic value of both LV GLS and RV FWS in a large cohort of patients with severe AS and referred for transcatheter aortic valve implantation (TAVI).

## 2. Materials and Methods

### 2.1. Patient Population and Data Collection

Patients referred for a TAVI between November 2007 and December 2019 were included from an ongoing registry of patients with severe AS at the Leiden University Medical Centre, the Netherlands. Severe AS was defined as an aortic valve area < 1 cm^2^ (or indexed aortic valve area < 0.6 cm^2^/m^2^) and/or a mean aortic valve gradient ≥ 40 mmHg, and/or peak aortic jet velocity ≥ 4 m/s [20,21]. Patients with a previous aortic valve surgery or incomplete clinical and/or echocardiographic data were excluded. Baseline demographic and clinical variables, including cardiovascular risk factors, comorbidities, New York Heart Association (NYHA) functional class, and medications, were collected from the medical records. Chronic kidney disease was defined as an estimated glomerular filtration rate < 60 mL/min/m^2^. The Institutional Review Board approved this retrospective analysis and waived the need for written informed consent.

### 2.2. Echocardiography

Transthoracic echocardiography was performed before TAVI, using a commercially available system (VIVID 7, E9 and E95; GE-Vingmed, Horten, Norway). Echocardiographic images were stored for off-line analysis (EchoPac V. 204; GE-Vingmed, Horten Norway). M-mode, two-dimensional and color, continuous- and pulsed-wave Doppler images were obtained from the parasternal, apical, and subcostal views, according to the current guidelines [20].

From the parasternal long-axis view, LV dimensions were assessed, and LV mass was calculated using the Du Bois formula and indexed for body surface area. LV volumes were obtained from the apical two- and four-chamber views, and LVEF was calculated using the biplane Simpson’s method and indexed for body surface area [20]. The left atrial end-systolic volume was obtained from the apical two- and four-chamber views using Simpson’s method of disks and was indexed for body surface area [20].

LV filling pressures were estimated using the E/e’ ratio, with e’ representing the average value of both septal and lateral sides obtained from tissue Doppler imaging of the mitral annulus on the apical four-chamber view [22].

Pulmonary artery systolic pressure was calculated according to the Bernoulli equation, derived from the tricuspid regurgitation jet peak velocity and the estimated right atrial pressure, and was derived from inferior vena cava diameter and collapsibility. To assess the right ventricular systolic function, M-mode was used to measure tricuspid annular plane systolic excursion [20].

Finally, aortic, mitral, and tricuspid regurgitation severity were graded as none/mild, moderate, or severe according to current recommendations using an integrative approach that includes qualitative, semi-quantitative, and quantitative parameters [23]. In patients with atrial fibrillation, the measurements were averaged over three consecutive cardiac cycles [23].

### 2.3. Speckle-Tracking Echocardiographic Examination

The LV GLS and RV FWS were measured offline by two-dimension, speckle-tracking echocardiography using dedicated LV and RV software (EchoPac V.204 GE-Vingmed Ultrasound, Horten, Norway). 

The LV GLS was calculated using images from the apical four-, three- and two-chamber views zoomed on the LV at a frame rate of ≥50 frames/s. The LV endocardial border was automatically traced (with manual corrections if necessary) and tracked by the software through the cardiac cycle. The LV GLS was derived by averaging all segmental peak strain values from all apical views and was expressed as absolute values [20].

RV FWS was calculated using images from the RV-focused apical four-chamber view at a frame rate of ≥50 frames/s. The RV endocardial border was traced using the automatic RV wall-detection algorithm. Tracing (with manual corrections if necessary) and tracking quality during the cardiac cycle were verified. The RV FWS was derived by averaging the three segments of the RV free wall and expressed as absolute values [24,25].

### 2.4. Follow Up and Outcome

The primary outcome of this study was all-cause mortality. Data on all-cause mortality were obtained from the departmental cardiology information system (EPD-Vision 12.9.9.3), which is directly linked to the governmental death registry database and therefore complete for all patients. 

### 2.5. Statistical Analysis

Categorical data are presented as absolute numbers and percentages. Continuous data are presented as mean ± standard deviation (SD) if normally distributed or as median (inter-quartile range, IQR) if not normally distributed. An analysis of variance with Bonferroni’s post hoc analysis or Kruskal–Wallis test for normally and non-normally distributed variables, respectively, was used to compare continuous variables between groups. The Pearson chi-square test was used to compare categorical variables.

The thresholds for dichotomizing LV GLS and RV FWS were based on previously published data, with cut-off values of LV GLS < 15% and RV FWS ≤ 20% to define impaired LV and RV systolic function, respectively [5,25]. The values above these cut-offs were defined as preserved chamber functions. In addition, the representability of the cut-off values in the current study population was tested with a fitted spline curve analysis. For this analysis, the estimated hazard ratio (HR) changes for all-cause mortality across the range of LV GLS and RV FWS values associated with an increased risk of all-cause mortality (i.e., predicted HR > 1) were used to define impaired LV and RV function, respectively. 

The patients were divided into one of the following three groups, according to the presence of impaired LV GLS (cut-off value < 15%) or impaired RV FWS (cut-off value ≤ 20%): (1) preserved strain group: referred to patients with preserved LV GLS and preserved RV FWS, (2) Single-ventricle impaired strain group: referred to patients with either impaired LV GLS or impaired RV FWS, (3) biventricular-impaired strain group: referred to patients with impaired LV GLS and impaired RV FWS.

Cumulative event-free survival was estimated using the Kaplan–Meier survival analysis with log-rank test, stratified by the three strain-based groups.

Cox proportional hazards regression analysis was performed to investigate the association between clinical and echocardiographic parameters with all-cause mortality. Variables in the univariable Cox regression analysis with *p* < 0.05 were considered statistically significant and were included in the multivariable Cox regression analysis. The baseline model included clinical and conventional echocardiographic parameters. Additionally, the strain-based groups were added to the baseline model and association with outcomes was evaluated.

For uni- and multivariable analyses, HR and 95% confidence interval (CI) were represented. Collinearity between all pairs of continuous variables included in the multivariable analysis was tested by a correlation factor analysis (correlation coefficient < 0.7).

To investigate the incremental value of the strain-based groups over the baseline model in association with outcome, a likelihood ratio test was performed and the change in global χ^2^ values was calculated and reported.

Additionally, a sensitivity analysis was performed in patients with LVEF ≥ 50%. Similar to the previously described approach, a Kaplan–Meier survival analysis stratified by the strain-based groups was performed, and an estimated five-year survival was reported. Furthermore, the association with all-cause mortality was tested by Cox regression analysis. The multivariable analysis included statistically significant variables (*p* < 0.05) on the univariable analysis.

Twenty patients were randomly selected for the evaluation of the intra-observer and inter-observer variability of LVGLS and RVFWS. Excellent agreement was defined by an intra-class correlation coefficient > 0.90, whereas good agreement was defined by a value between 0.75 and 0.90. All hypothesis tests had a two-sided significance level of <0.05. Statistical analysis was performed using SPSS for Windows, version 29.0 (IBM Armonk, NY, USA) and R version 4.2.1 (R Foundation for Statistical Computing, Vienna, Austria).

## 3. Results

### 3.1. Patient Population

**Figure 1:**
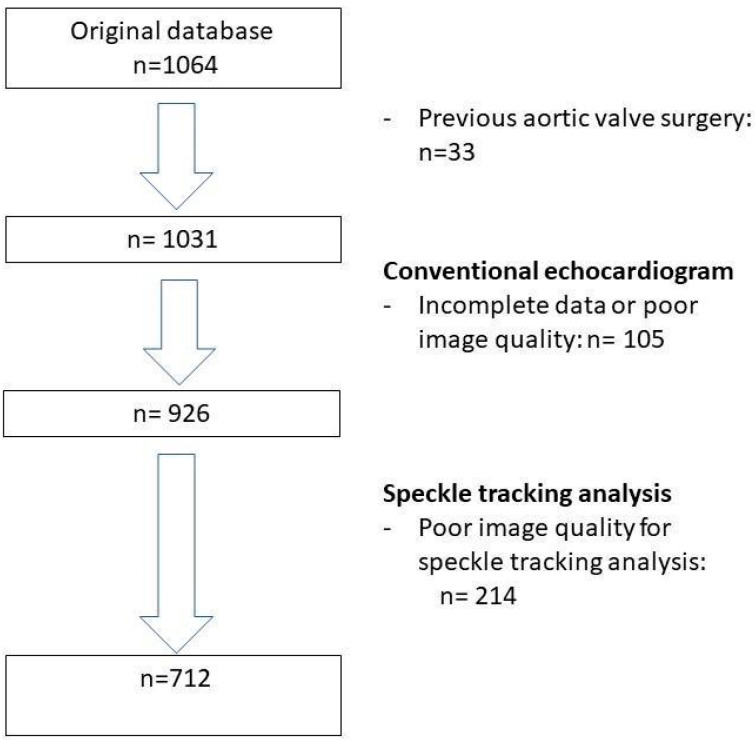
Flowchart of the study population.

A total of 712 patients (mean age 80 ± 7 years, 53% men) were included from a cohort of 1064 patients who underwent TAVI for severe AS at the Leiden University Medical Center, the Netherlands (Figure 1). The majority of patients had several cardiovascular risk factors including arterial hypertension (74%) and dyslipidemia (63%). More than half of the patients (59%) had coronary artery disease, of whom 18% had a previous coronary artery bypass graft surgery (Table 1). Table 2 displays the baseline echocardiographic characteristics of the total population, including valvular and ventricular abnormalities.

### 3.2. Follow up and Outcome

During a median follow up of 52 (IQR: 34–73) months, 248 (35%) patients died. The overall mortality rate was 21% at 3 years, and 34% at 5 years. 

**Figure 2:**
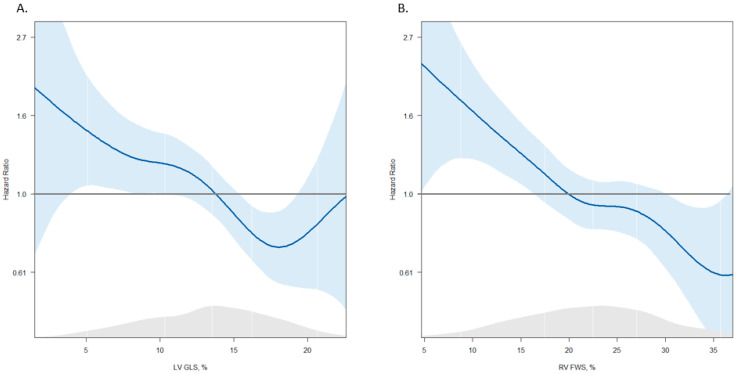
Spline curves for all-cause mortality according to LV GLS (**A**) and RV FWS (**B**). The spline curves describe the HR change for the primary endpoint with 95% CI (shaded blue areas) across the range of values of LV GLS (**A**) and RV FWS (**B**). The HR starts to increase and exceeds the HR of one for LV GLS < 15% (**A**) and for RV FWS ≤ 20% (**B**). LV GLS: left ventricular global longitudinal strain; RV FWS: right ventricular free wall strain; HR: hazard ratio; CI: confidence interval.

A spline curve was fitted to evaluate the association between LV GLS and RV FWS with all-cause mortality. With decreasing values of LV GLS and RV FWS, the HR for the primary endpoint increased. The HR exceeded the threshold of >1 for LV GLS < 15% and RV FWS ≤ 20% (Figure 2). These thresholds were concordant with previously published data and were used to stratify the population in the three strain-based groups [5,25]. 

The correlation coefficient between LV GLS and RV FWS was 0.38.

**Figure 3:**
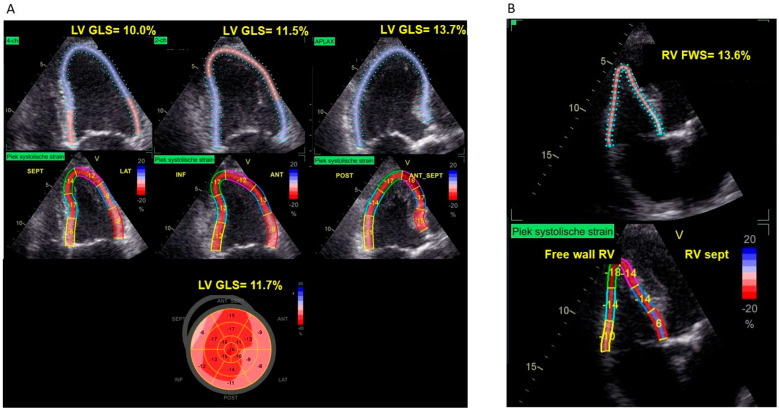
Example of LV GLS and RV FWS measurement in a patient with severe AS who died during follow up. (**A**) Echocardiographic images of LV GLS from four-, two-, and three-chamber views with bull’s eye plot. The bull’s eye plot demonstrates an impairment of LV GLS (11.7%), particularly of the basal LV segments. (**B**) Echocardiographic images of RV FWS from the RV-focused apical four-chamber view. The absolute value of 13.6% of RV FWS demonstrates an impaired RV FWS. LV: left ventricle; LV GLS: LV global longitudinal strain; RV: right ventricle; RV FWS: RV free wall strain; AS: aortic stenosis. Strain values are expressed as absolute values. 4-ch: 4-chamber view; 2-ch: 2-chamber view; APLAX: apical long axis view. TAPSE: tricuspid annular plane systolic excursion; ANT: anterior; ANT_SEPT: antero-septal; INF: inferior; LAT: lateral; POST: posterior; SEPT: septal.

There were 191 patients (27%) in the preserved strain group, 314 patients (44%) in the single-ventricle impaired group, and 207 patients (29%) in the biventricular-impaired strain group. Of the patients in the single-ventricle impaired strain group, 81% had impaired LV GLS only, while 19% of them had impaired RV FWS only. Figure 3 demonstrates an example of a patient in the biventricular-impaired strain group, with both reduced LV GLS and RV FWS, who died during follow-up.

Regarding the clinical characteristics (Table 1), significant differences were observed between groups for sex (being male more represented in the biventricular-impaired strain group), body surface area, coronary artery disease and coronary artery bypass graft surgery, renal function, atrial fibrillation, severe symptoms (i.e., NYHA class III-IV), and use of diuretics.

Regarding the echocardiographic characteristics (Table 2), patients in the single-ventricle impaired strain and biventricular-impaired strain group had higher LV volumes and more hypertrophic remodeling as compared to the preserved strain group. Approximately one of four patients (27%) had LVEF < 50% in the single-ventricle impaired strain group, while 64% of patients in the biventricular-impaired strain group had LVEF < 50%. According to the group definition, LV GLS was progressively lower in the single-ventricle impaired strain and biventricular-impaired strain group (as compared to the preserved strain group, 13 ± 3% and 10 ± 3% vs. 18 ± 2%, *p* < 0.001). Similar for RV FWS, progressively lower values were observed in the single-ventricle impaired strain and biventricular-impaired strain group (as compared to the preserved strain group; 24 ± 5% and 15 ± 4% vs. 28 ± 5%, *p* < 0.001, respectively). 

The parameters representing LV diastolic dysfunction were affected in all groups. However, values for left atrial volume index, filling pressures, and systolic pulmonary arterial pressure were significantly higher in the biventricular-impaired strain group as compared to the preserved strain group.

Finally, aortic valve area did not differ between the groups and concomitant severe aortic, mitral, or tricuspid regurgitation was observed only in few patients (2%, 6% and 5%, respectively).

### 3.3. Survival Analysis According to Ventricular Functions

**Figure 4:**
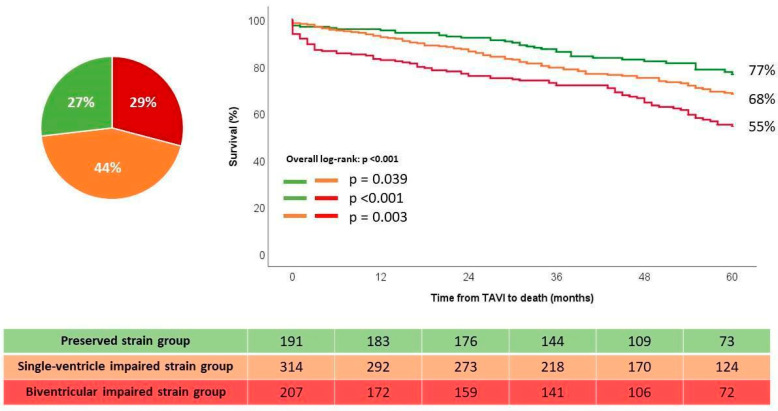
Kaplan–Meier estimated survival curves according to the strain-based groups. TAVI: transcatheter aortic valve implantation.

The Kaplan–Meier survival analysis showed that patients in the single-ventricle impaired strain and biventricular-impaired strain groups had significantly lower estimated cumulative survival rates at three- and five-years follow up, as compared to the preserved strain group (79% and 68% for the single-ventricle impaired strain group; 73% and 55% for the biventricular-impaired strain group; vs. 87% and 77% for the preserved strain group, respectively, *p* < 0.001, Figure 4). In the single-ventricle impaired strain group, no difference in survival was noted between the group with impaired LV GLS and preserved strain RV FWS vs. the group with preserved LV GLS and impaired RV FWS (Supplementary Figure S1).

At the univariable Cox regression analysis (Table 3), several clinical characteristics were significantly associated with all-cause mortality. Among the echocardiographic characteristics, significant association with the primary endpoint (*p* < 0.05) was observed for LVEF < 50%, severe mitral regurgitation, severe tricuspid regurgitation, and strain-based groups (Table 3).

For a multivariable analysis, a baseline model was built with the following clinical and echocardiographic variables, significant of an univariable regression analysis: age, sex, smoking, diabetes mellitus, coronary artery disease, peripheral artery disease, chronic kidney disease, NYHA functional class III-IV, LVEF < 50%, severe mitral regurgitation, and severe tricuspid regurgitation. From this model, only male sex, smoking, chronic kidney disease, and severe tricuspid regurgitation remained independently associated with outcome. After adding the strain-based groups to this baseline model, an independent association between the strain-based groups and all-cause mortality was observed together with male sex, smoking, and chronic kidney disease. In particular, there was an increasing HR for the single-ventricular impaired strain group (HR: 1.716; 95% CI (1.084–2.117), *p* = 0.021) and the biventricular-impaired strain group (HR: 1.902; 95% CI (1.116–3.241), *p* = 0.018) as compared to the preserved strain group (reference group, overall *p*-value = 0.040) (see Table 4).

**Figure 5:**
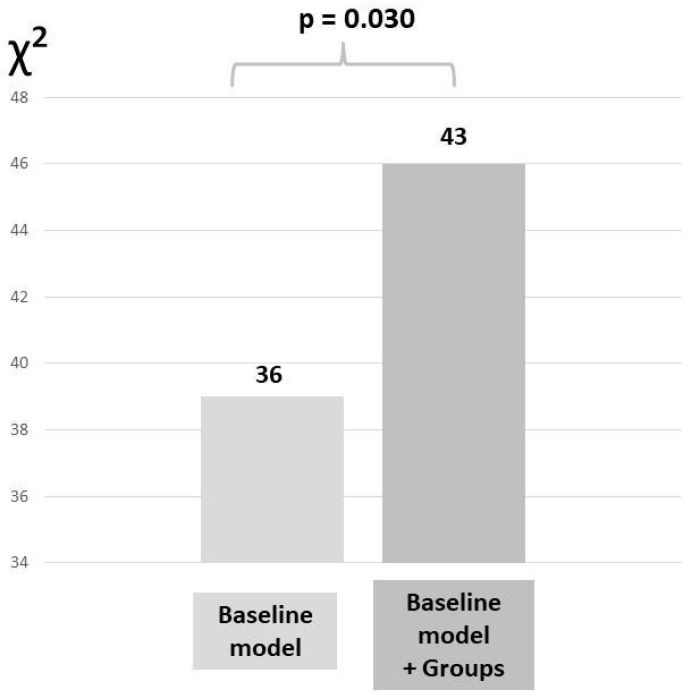
Likelihood ratio test for the incremental value of adding strain-based groups to the baseline model to evaluate the association with all-cause mortality. The baseline model included: age, sex, smoking, diabetes mellitus, coronary artery disease, peripheral artery disease, chronic kidney disease, history of atrial fibrillation, New York Heart Association functional class III or IV, left ventricular ejection fraction < 50%, severe mitral regurgitation, and severe tricuspid regurgitation.

Additionally, a likelihood ratio test was performed to determine the incremental value of the strain-based groups over the baseline model. The addition of the strain-based group to the baseline model resulted in a significant increase in the χ^2^ value (χ^2^ difference = 7, *p* = 0.030), demonstrating the incremental value of this biventricular assessment to classify patients with severe AS undergoing TAVI (Figure 5).

### 3.4. Sensitivity Analysis in Preserved Left Ventricular Ejection Fraction

**Figure 6:**
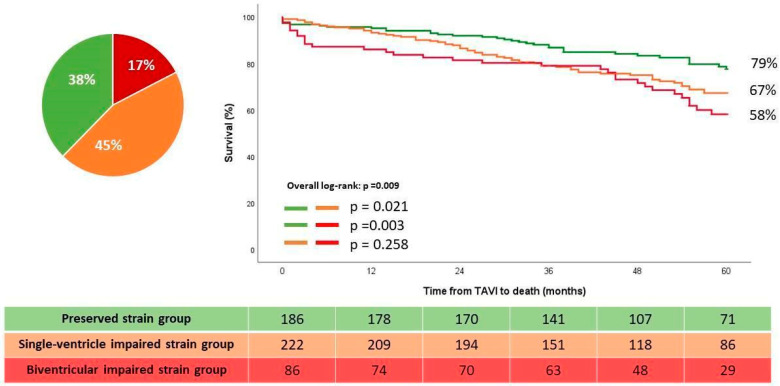
Kaplan–Meier estimated survival curves according to the strain-based groups in patients with LVEF ≥ 50%. LVEF: Left ventricular ejection fraction; TAVI: transcatheter aortic valve implantation.

Further sensitivity analysis was performed in patients with preserved LVEF (i.e., LVEF ≥ 50%). Of the 494 patients, 155 (31%) patients died during a median follow up of 52 months (IQR: 34–73 months). The Kaplan–Meier survival analysis showed a significant difference in estimated the five-years survival rates between the single-ventricle impaired strain and biventricular-impaired strain group as compared to the preserved strain group (67% and 58% vs. 79%, overall log-rank test *p* = 0.009) (Figure 6). On the uni- and multivariate Cox regression analysis, the single-ventricle impaired strain and biventricular impaired strain group (with the preserved strain group as reference) remained significantly and independently associated with the primary endpoint (Table 5).

### 3.5. Reproducibility 

Intra-observer and inter-observer variability both showed excellent agreements, with an intra-class correlation coefficient, respectively, of 0.988 (95% CI 0.969 to 0.995) and 0.932 (95% CI 0.925 to 0.981) for LV GLS and of 0.903 (95% CI 0.756 to 0.961) and 0.905 (95% CI 0.888 to 0.964), respectively, for RVFWS.

## 4. Discussion

In this large cohort of patients with severe AS referred for TAVI, the prognostic importance of biventricular strain assessment was evaluated. The main findings are as follows: (1) both LV and RV strain measurements were superior to conventional echocardiographic measurements and were independently associated with all-cause mortality, (2) mortality risk increased progressively when the strain of one or both ventricular (LV/RV) chambers was impaired and (3) similar results were observed in patients with preserved LVEF.

### 4.1. LV GLS and RV FWS as Markers of Subclinical Dysfunction and Prognosis in Patients with Severe AS

In severe AS, increased afterload induces concentric LV remodeling in order to compensate for the increased LV wall stress. However, over time, especially when progressive myocardial fibrosis occurs, this adaptive mechanism may fail and lead to LV dysfunction. LV function deterioration typically affects first LV longitudinal contraction, reflected by an impairment of the LV longitudinal strain. In AS patients, LV GLS has been associated with the severity of myocardial fibrosis and has been shown to be a more sensitive marker for LV dysfunction than LVEF, since its impairment precedes the reduction in LVEF [26,27].

A recent meta-analysis showed that an impaired baseline LV GLS was associated with a significantly higher post-TAVI risk for all-cause mortality and with an incremental value over the conventional echocardiographic parameters [5]. Even in asymptomatic or only mildly symptomatic patients with preserved LVEF and severe AS, LV GLS showed prognostic importance for risk stratification [28,29].

The hemodynamic effects of chronic pressure overload due to AS are not limited to the LV. Post-capillary pulmonary hypertension due to elevated LV filling pressures, and possibly concomitant mitral regurgitation, can lead to secondary tricuspid regurgitation, RV dilatation, and eventually RV dysfunction [10,12,30]. Because of the complex RV geometry and physiology, conventional echocardiographic parameters are limited in the assessment of RV remodeling and function [16]. Medvedofsky et al. showed that in patients with severe AS, the degree of RV function as assessed by RV FWS, rather than conventional RV function parameters, was a major determinant of 1-year mortality post TAVI [13].

### 4.2. Incremental Value of Biventricular Strain for Risk Stratification in Patients with Severe AS

Few studies have evaluated the incremental prognostic value of a biventricular strain assessment in patients with severe AS [17,18,19]. In a cohort of 128 patients with severe low-flow, low-gradient AS, and after the exclusion of more than mild left-sided valve disease, Dahou et al. demonstrated that both LV GLS and RV FWS are independent predictors of mortality. Furthermore, in this high-risk subgroup of low-flow, low-gradient AS patients, both LV GLS and RV FWS showed an incremental prognostic value of known demographic and echocardiographic predictors of outcomes [18]. These findings were confirmed and extended in the present study with a larger population and, importantly, included the complete spectrum of AS subtypes.

Similarly, Ye et al. implemented a multi-chamber, strain-based staging model including the left atrium, LV and RV strain, in patients with more than moderate AS in whom aortic intervention (surgical or transcatheter) was performed with 56% of patients having tricuspid and 85% of patients having bicuspid AS. Multi-chamber, strain-based staging was independently associated with all-cause mortality with increasing risk per stage, and provided additional value in risk stratification compared to the conventional echocardiographic staging approach [1,6,19,31]. The present study confirmed the incremental value of and the association of biventricular strain with all-cause mortality in a homogeneous population with severe AS who underwent TAVI.

Conversely, in a selected cohort of 100 patients with severe AS referred for TAVI, only RV FWS but not LV GLS was associated with cardiovascular mortality [17]. In comparison with the present study, those patients probably presented with a more advanced stage of AS disease since they had significantly lower values of LV GLS and RV FWS (11% and 18% vs. 13% and 22%, respectively) [17].

Of interest, in the current study, a group of patients with preserved LV GLS but impaired RV FWS was identified possibly due to underlying primary RV pathology or pulmonary vascular disease. This subgroup was characterized by higher mortality rates, as compared to the preserved strain group, but lower mortality rates as compared to patients with biventricular impairment.

Of note, biventricular strain measurement may be influenced by sex differences, as men and women have shown different chamber remodeling in response to aortic stenosis [32,33]. In the current study, (male) sex was significantly and independently associated with outcomes together with the strain-based groups. Further research could explore the potential implications of sex differences in biventricular strain.

### 4.3. Clinical Implications

The current study shows that assessing both LV and RV strain may help detect subclinical myocardial dysfunction and may improve risk stratification in patients with severe AS referred for TAVI. Since current guidelines recommend interventions only in symptomatic patients or in asymptomatic patients with reduced LVEF, assessment of biventricular strain could be considered to improve selection of patients at higher risk for adverse events, who may require close follow up and may benefit from earlier valve intervention.

### 4.4. Limitations

This study was limited by its retrospective design and the findings need to be confirmed in a prospective, multi-center setting. Patients with incomplete echocardiographic biventricular strain data were excluded, which may have created selection bias. However, as shown in the supplemental Table S1, patients included in the study had similar clinical and echocardiographic characteristics as compared to the ones excluded. Also, one echocardiographic vendor was used for strain assessment, and the current cut-off values applied to define strain impairment might not be applicable to other echocardiography vendors.

## 5. Conclusions

In patients with severe AS undergoing TAVI, biventricular strain impairment is associated with an increased risk of all-cause mortality post TAVI, and may improve risk stratification, particularly in patients with preserved LVEF.

## Figures and Tables

**Table 1:** Baseline demographic characteristics of the total study population and per strain-based group.

	Total Population n = 712	Preserved Strain Group n = 191	Single-Ventricle Impaired Strain Group n = 314	Biventricular-Impaired Strain Group n = 207	*p*-Value
**Age, years**	80 (±7)	80 (±7)	80 (±7)	79 (±7)	0.229
**Male sex, n (%)**	377 (53)	83 (44)	148 (47)	146 (71) * ^+^	**<0.001**
**BSA, m^2^**	1.9 (±0.2)	1.8 (±0.2)	1.8 (±0.2)	1.9 (±0.2) * ^+^	**<0.001**
**Smoking, n (%)**	153 (23)	37 (21)	64 (22)	52 (27)	0.361
**Arterial hypertension, n (%)**	527 (74)	150 (79)	227 (73)	150 (73)	0.234
**Diabetes mellitus, n (%)**	201 (28)	52 (27)	91 (21)	58 (28)	0.931
**Dyslipidemia, n (%)**	447 (63)	122 (64)	193 (62)	132 (64)	0.794
**Coronary artery disease, n (%)**	421 (59)	96 (51)	193 (62) *	132 (64) *	**0.015**
**Previous CABG, n (%)**	114 (18)	18 (11)	44 (15)	52 (31) * ^+^	**<0.001**
**Peripheral artery disease, n (%)**	208 (29)	52 (27)	96 (31)	60 (29)	0.724
**Chronic kidney disease, n (%)**	209 (30)	46 (24)	84 (27)	79 (39) * ^+^	**0.003**
**Atrial fibrillation, n (%)**	169 (24)	20 (11)	69 (22) *	80 (39) * ^+^	**<0.001**
**NYHA class III-IV, n (%)**	403 (57)	91 (48)	178 (56)	134 (65) * ^+^	**0.003**
**Betablockers, n (%)**	417 (59)	104 (55)	196 (62)	117 (57)	0.189
**Diuretics, n (%)**	394 (55)	74 (39)	175 (56) *	145 (70) * ^+^	**<0.001**
**RAAS-inhibitors, n (%)**	376 (53)	98 (52)	162 (52)	116 (56)	0.520
**Statins, n (%)**	452 (64)	121 (64)	204 (65)	127 (62)	0.744

Values are expressed as mean ± SD, median (IQR) or n (%). Abbreviations: BSA: Body Surface Area; CABG: coronary artery bypass graft; NYHA: New York Heart Association; RAAS-inhibitors: Renin- angiotensin-aldosterone system inhibitors. Chronic kidney disease was defined as an estimated glomerular filtration rate < 60 mL/min/m^2^. * Significant difference with “preserved strain group”; ^+^ Significant difference with “single-ventricle impaired strain group” (after Bonferroni correction).

**Table 2:** Baseline echocardiographic characteristics of the total study population and per strain-based group.

	Total Population n = 712	Preserved Strain Group n = 191	Single-Ventricle Impaired Strain Group n = 314	Biventricular-Impaired Strain Group n = 207	*p*-Value
**LV end–diastolic volume index, mL**	55 (±24)	46 (±14)	53 (±21) *	65 (±31) * ^+^	**<0.001**
**LV end–systolic volume index, mL**	26 (±19)	17 (±8)	25 (±16) *	37 (±24) * ^+^	**<0.001**
**LV ejection fraction < 50%**	218 (31)	0	85 (27) *	133 (64) * ^+^	**<0.001**
**LV mass index, g/m^2^**	125 (±38)	114 (±34)	127 (±36) *	133 (±39) *	**<0.001**
**LV global longitudinal strain, %**	13 (±4)	18 (±2)	13 (±3) *	10 (±3) * ^+^	**<0.001**
**Left atrial volume index, mL/m^2^**	41 (31–53)	38 (29–46)	40 (31–52)	46 (36–57) * ^+^	**<0.001**
**E/e’ ratio**	17 (12–24)	15 (12–21)	17 (12–24)	19 (14–26) *	**0.007**
**Severe mitral regurgitation**	40 (6)	4 (2)	18 (6)	18 (9) *	**0.019**
**Aortic valve area, cm^2^**	0.8 (±0.3)	0.8 (±0.3)	0.8 (±0.3)	0.8 (±0.3)	0.602
**Mean aortic valve gradient, mmHg**	42 (±16)	46 (±17)	44 (±16)	36 (±15) * ^+^	**<0.001**
**Peak aortic velocity (m/s)**	4 (±0.7)	4 (±0.5)	4 (±0.4)	4 (±0.5) * ^+^	**<0.001**
**Severe aortic regurgitation**	17 (2)	4 (2)	6 (2)	7 (3)	0.300
**TAPSE, mm**	19 (±5)	21 (±4)	19 (±4) *	16 (±4) * ^+^	**<0.001**
**RV free wall strain, %**	22 (±7)	28 (±5)	24 (±5) *	15 (±4) * ^+^	**<0.001**
**PASP, mmHg**	35 (29–44)	30 (35–42)	33 (27–42)	38 (30–49) * ^+^	**0.004**
**Severe tricuspid regurgitation**	33 (5)	3 (2)	10 (3)	20 (10) *	**<0.001**

Values are expressed as mean ± SD, median (IQR) or n (%). Abbreviations: LV: left ventricle; TAPSE: tricuspid annular plane systolic excursion; RV: right ventricle; PASP: pulmonary artery systolic pressure. * Significant difference with “preserved strain group”; ^+^ Significant difference with “single-ventricle impaired strain group” (after Bonferroni correction).

**Table 3:** Univariable Cox proportional hazard analysis for all-cause mortality.

	HR (95% CI)	*p*-Value
**Age**	**1.255 (1.050–1.772)**	**0.029**
**Male sex**	**1.539 (1.193–1.987)**	**<0.001**
**Smoking**	**1.720 (1.313–2.253)**	**<0.001**
**Arterial hypertension**	1.074 (0.805–1.434)	0.627
**Diabetes mellitus**	**2.024 (1.499–2.732)**	**<0.001**
**Dyslipidemia**	1.198 (0.920–1.560)	0.180
**Coronary artery disease**	1.407 (1.059–1.870)	0.019
**Peripheral artery disease**	**1.738 (1.348–2.240)**	**<0.001**
**Chronic kidney disease**	1.609 (1.225–2.114)	<0.001
**Atrial fibrillation**	1.292 (0.972–1.719)	0.078
**NYHA III-IV**	**1.131 (1.010–1.267)**	**0.033**
**LVEF < 50%**	**1.421 (1.067–1.892)**	**0.016**
**LV mass index**	0.947 (0.997–1.003)	0.947
**Left atrial volume index**	1.005 (0.997–1.012)	0.201
**Severe mitral regurgitation**	**1.459 (1.070–2.099)**	**0.017**
**Severe aortic regurgitation**	1.237 (0.915–1.674)	0.167
**TAPSE**	0.981 (0.952–1.011)	0.215
**PASP**	1.011 (0.999–1.022)	0.063
**Severe tricuspid regurgitation**	**1.809 (1.070–3.058)**	**0.027**
**Strain-based groups:**		**<0.001**
**Preserved**	**Reference group**	
**Single-ventricle impaired**	**1.477 (1.010–2.160)**	**0.037**
**Biventricular impaired**	**2.310 (1.575–3.385)**	**<0.001**

Abbreviations: HR: hazard ratio; CI: confidence interval; NYHA: New York Heart Association; LVEF: left ventricular ejection fraction; TAPSE: tricuspid annular plane systolic excursion; PASP: pulmonary artery systolic pressure. Age is expressed per 5-year increase. Chronic kidney disease was defined as an estimated glomerular filtration rate < 60 mL/min/m^2^.

**Table 4:** Multivariable Cox proportional hazard analysis for all-cause mortality.

	Baseline Model		Baseline Model + Strain Groups	
	HR (95% CI)	*p*-Value	HR (95% CI)	*p*-Value
**Age**	0.963 (0.874–1.061)	0.446	0.964 (0.874–1.063)	0.464
**Male sex**	1.583 (1.113–2.251)	0.011	1.528 (1.071–2.180)	0.019
**Smoking**	1.475 (1.026–2.121)	0.036	1.439 (1.008–2.069)	0.040
**Diabetes mellitus**	1.038 (0.729–1.477)	0.838	1.013 (0.711–1.443)	0.942
**Coronary artery disease**	1.271 (0.880–1.836)	0.064	1.230 (0.849–1.782)	0.073
**Peripheral artery disease**	1.372 (0.973–1.933)	0.071	1.412 (1.002–1.990)	0.079
**Chronic kidney disease**	1.455 (1.046–2.024)	0.026	1.411 (1.036–2.003)	0.030
**History of atrial fibrillation**	1.116 (0.765–1.627)	0.301	1.116 (0.765–1.627)	0.570
**NYHA III-IV**	1.229 (0.877–1.722)	0.231	1.186 (0.847–1.662)	0.321
**LVEF < 50%**	0.915 (0.620–1.351)	0.655	0.787 (0.525–1.179)	0.245
**Severe mitral regurgitation**	1.147 (0.587–2.244)	0.188	1. 060 (0.542–2.074)	0.865
**Severe tricuspid regurgitation**	1.493 (0.735–3.032)	0.026	1.412 (1.002–1.990)	0.331
**Strain-based groups:**				0.040
**Preserved**			Reference group	
**Single-ventricle impaired**			1.716 (1.084–2.717)	0.021
**Biventricular impaired**			1.902 (1.116–3.241)	0.018

Abbreviations: HR: hazard ratio; CI: confidence interval; CABG: coronary artery bypass graft; NYHA: New York Heart Association; LVEF: LV ejection fraction. Age is expressed per 5-year increase, CKD was defined as an estimated glomerular filtration rate < 60 mL/min/m^2^.

**Table 5:** Cox proportional hazard analysis for all-cause mortality in patients with LVEF ≥ 50%.

Univariable Analysis			Multivariable Analysis *	
Variable	HR (95% CI)	*p*-Value	HR (95% CI)	*p*-Value
**Strain-based groups**		0.011		0.033
**Preserved**	Reference group		Reference group	
**Single-ventricle impaired**	1.608 (1.065–2.427)	0.024	1.872 (1.169–3.136)	0.010
**Biventricular impaired**	2.050 (1.264–3.324)	0.004	2.018 (1.068–3.639)	0.030

Abbreviations: LVEF: left ventricular ejection fraction; HR: hazard ratio; CI: confidence interval. * Adjusted for: age, male sex, smoking, diabetes mellitus, coronary artery disease, peripheral artery disease, chronic kidney disease, atrial fibrillation, severe tricuspid regurgitation.

## Data Availability

Data are available upon reasonable request.

## Supplementary Materials

The following supporting information can be downloaded at: https://www.mdpi.com/article/10.3390/jcdd11030090/s1, Figure S1: Kaplan–Meier estimated survival curves for the single-ventricle impaired strain group according to LV GLS or RV FWS impairment. Table S1: Baseline clinical and echocardiographic characteristics of the included and excluded patients.
