# Supplementary material for: Incremental Value of Biventricular Strain in Patients with Severe Aortic Stenosis
Source: J Cardiovasc Dev Dis. 2024 Mar 13;11(3):90. doi: 10.3390/jcdd11030090 (PMC10971233; doi:10.3390/jcdd11030090)
Supplement: Supplementary file 1 [file jcdd-11-00090-s001.zip › jcdd-2890102-supplementary.pdf]

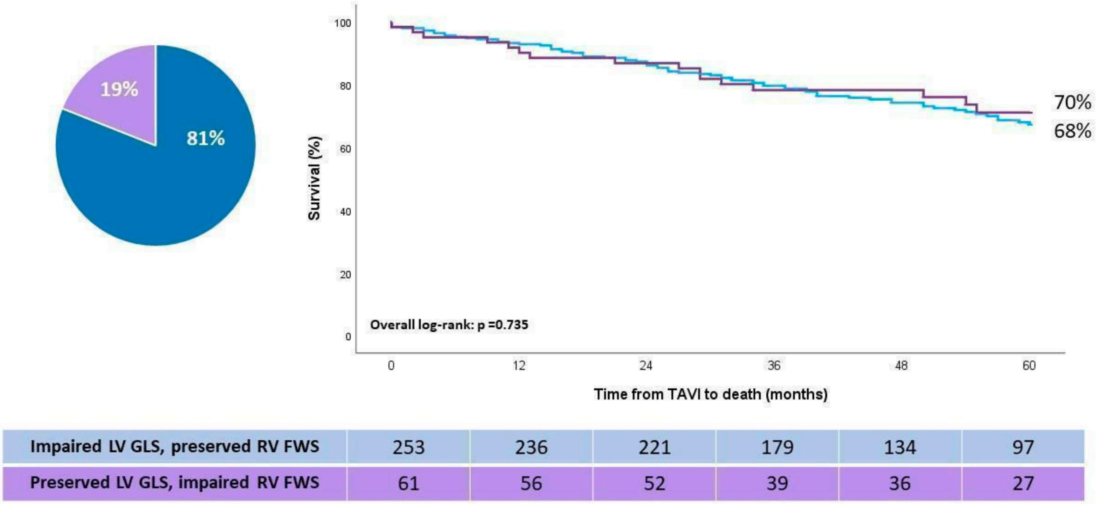

Supplementary Figure S1. Kaplan-Meier estimated survival curves for the single-ventricle impaired strain group according to LV GLS or RV FWS impairment.

LV GLS: Left ventricular global longitudinal strain; RV FWS: Right ventricular free wall strain; TAVI: transcatheter aortic valve implantation.

Supplementary Table S1. Baseline clinical and echocardiographic characteristics of the included and excluded patients.

	**Included patients n=712**	**Excluded patients n=319**	**p- value**
Age, years	**80 (±7)**	**79 (±7)**	**0.041**
Male sex, n (%)	377 (53)	177 (55)	0.556
BSA, m^2	**1.9 (±0.2)**	**1.8 (±0.2)**	**0.005**
Smoking, n (%)	153 (23)	65 (20)	0.404
Arterial hypertension, n (%)	527 (74)	241 (75)	0.879
Diabetes mellitus, n (%)	201 (28)	99 (31)	0.557
Dyslipidemia, n (%)	447 (63)	213 (67)	0.579
Coronary artery disease, n (%)	421 (59)	194 (61)	0.787
Peripheral artery disease, n (%)	208 (29)	85 (27)	0.237
Chronic kidney disease, n (%)	52 (7)	56 (8)	0.394
Atrial fibrillation, n (%)	169 (24)	94 (29)	0.066
NYHA class III-IV, n (%)	403 (57)	204 (64)	0.055

LV end– diastolic volume index, mL	55 (±24)	54 (±24)/102 missings	0.399
LV end– systolic volume index, mL	26 (±19)	27(±18)/102 missings	0.635
LV ejection fraction, %	55 (±14)	54 (±14)/102 missings	0.023
LV mass index, g/m²	125 (±38)	129 (±42)	0.895
LV global longitudinal strain, %	13 (±4)	13 (±4)/194 missings	0.738
Left atrial volume index, mL/m²	41 (31 – 53)	41 (31 - 52)/99 missings	0.293
E/e’ ratio	17 (12 – 24)	17 (12 - 24)/98 missings	0.463
Severe mitral regurgitation	40 (6)	8 (3)	0.053
Aortic valve area, cm²	0.8 (±0.3)	0.8 (±0.3)	0.060
Severe aortic regurgitation	17 (2)	7 (2)	0.248
TAPSE, mm	19 (±5)	19 (±5)/103 missings	0.865
RV free wall strain, %	22 (±7)	20 (±6)/202 missings	0.080
PASP, mmHg	35 (29 – 44)	35 (29 - 44)/97 missings	0.916
Severe tricuspid regurgitation	33 (5)	11 (3)	0.892

Values are expressed as mean ±SD, median (IQR) or n (%).
Abbreviations: BSA: Body Surface Area; NYHA: New York Heart Association; LV: left ventricle, TAPSE: tricuspid annular plane systolic excursion; RV: right ventricle; PASP: pulmonary artery systolic pressure.
